# Canal Transportation and Centering Ability of ProTaper and SafeSider in Preparation of Curved Root Canals: A CBCT Evaluation

**DOI:** 10.22037/iej.v13i2.19200

**Published:** 2018

**Authors:** Negar Delgoshayi, Mansoure Abbasi, Hengameh Bakhtiar, Shirin Sakhdari, Setareh Ghannad, Mohammad Reza Ellini

**Affiliations:** a *Dental Material Research Center, Department of Endodontics, Tehran Dental Branch, Islamic Azad University, Tehran, Iran;*; b *Department of Endodontics, Tehran Dental Branch, Islamic Azad University, Tehran, Iran;*; c *Dental Material Research Center, Department of Endodontics, Tehran Dental Branch, Islamic Azad University, Tehran, Iran;*; d *Department of Radiology, Tehran Dental Branch, Islamic Azad University, Tehran, Iran*

**Keywords:** Canal Transportation, Cone-beam Computed Tomography, ProTaper, Root Canal Preparation, SafeSider

## Abstract

**Introduction:**

Maintaining the original central canal path is an important parameter in efficient root canal preparation. Instruments causing minimal changes in original canal path are preferred for this purpose. This study sought to compare canal transportation and centering ability of ProTaper and SafeSider instruments in curved mesiobuccal root canals of mandibular first molars using cone beam computed tomography (CBCT).

**Methods and Materials:**

In this experimental study, 30 mesiobuccal root canals of extracted human mandibular first molars with 20° to 40° curvature were randomly divided into two groups (*n*=15). After mounting in putty, preoperative CBCT scans were obtained of teeth. Root canals in group A were shaped using S1, S2, F1 and F2 of ProTaper system. Root canals in group B were instrumented to size 25 using SafeSider system according to the manufacturers’ instructions. Postoperative CBCT scans were then obtained. The distance between the external root surface and internal canal wall was measured at the mesial and distal at 1, 3 and 7 mm from the apex. The values measured on primary and secondary CBCT scans were compared to assess possible changes in original central canal path and canal transportation. Data were compared using the *t*-test and repeated measure ANOVA.

**Results:**

ProTaper and SafeSider were significantly different in terms of canal transportation and centering ability, and ProTaper was significantly superior to SafeSider in this respect (*P*<0.001).

**Conclusion:**

ProTaper (in contrast to SafeSider) is well capable of maintaining the original central canal path with the least amount of transportation.

## Introduction

Root canal preparation is performed to eliminate the inflamed pulp tissue and necrotic debris and shape the root canal system to enhance its irrigation and filling [1]. To achieve the best treatment outcome, conical preparation of root canal must follow the original central canal path. However, this goal is difficult to achieve particularly in curved root canals due to the tendency of files to regain their straight shape [2, 3]. This often leads to procedural errors such as transportation, ledge formation and perforation [4]. Several root canal preparation instruments have been introduced to prevent procedural errors. Nickel-titanium (NiTi) rotary files have high acceptance due to their high flexibility and optimal canal centering ability [1]. These systems have shorter working time and result in less procedural errors [5]. However, fatigue fracture of these instruments may occur particularly in curved and narrow canals [6, 7].

ProTaper universal system (Dentsply Maillefer, Ballaigues, Switzerland) files have a convex, triangular-shaped cross-section and a safe non-cutting tip. They have a shallow v-shaped groove, which according to the manufacturer, increases their flexibility. ProTaper system has a set of files with variable tapers. SX, S1 and S2 files have ascending tapers along their cutting blades. F1, F2 and F3 files have descending tapers [8]. 

**Figure 1:**
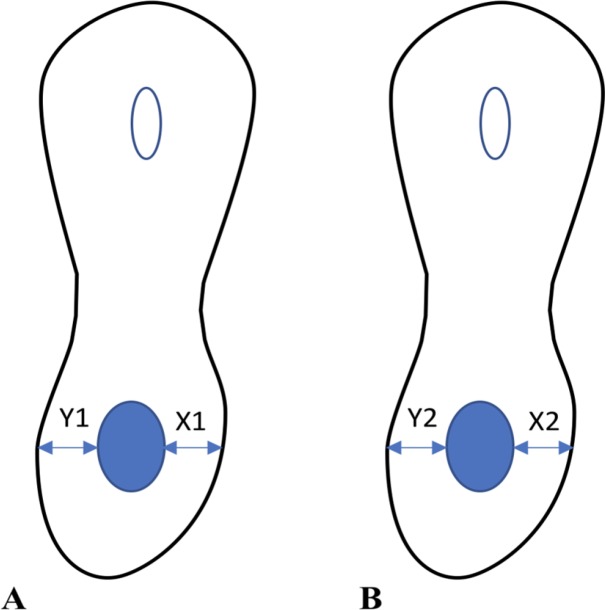
Schematic view of canal geometry *A)* Before; *B)* After preparation

These files have variable pitch and helical angle and their non-cutting tip decreases the possibility of canal transportation [8, 9]. Some studies reported that ProTaper system has a high canal centering ability [1, 8-11]. ProTaper system and other NiTi rotary files available in the market function based on continuous rotation movement [12, 13]. 

SafeSider system (Essential Dental Systems, South Hackensack, NJ, USA), with a reciprocating movement, has a flat-sided design, which decreases its engagement with dentin and increases its fracture strength during root canal preparation. SafeSider files are thinner and more flexible compared to other systems due to smaller cross-sectional diameter and flat sides. This system has eight stainless steel and three NiTi files [14]. However, several studies have reported that reciprocating motion increased the canal centering ability while it is considered to be similar to the manual balanced force technique [1, 8-11]. 

Several methods are available to assess canal centering ability of different root canal preparation systems such as the use of electron and light microscopy, micro-computed tomography, high resolution computed tomography and CBCT [15, 16]. In CBCT, three-dimensional images of an object are generated using a back filtered projection method. Although CBCT has lower spatial resolution than micro-computed tomography, the former provides valuable information about the morphology of root canals, fractures and changes in root structure [17].

Recent studies focus on improving NiTi rotary systems and recommend reciprocating movement to reduce instrument fracture [18, 19]. However, there is a limited knowledge on canal shaping ability of these instruments [20, 21]. The aim of this study is to compare the ProTaper (rotation system) and SafeSider (reciprocating movement) ability in preserving original root canal anatomy.

## Materials and Methods

This *in vitro *experimental study was conducted on 30 human mandibular first molars extracted due to periodontal reasons. The teeth were selected using convenience sampling. Sample size was calculated to be 30 samples based on previous studies [22]. The inclusion criteria were extracted mandibular first molars with closed apices, no internal or external root resorption, no root cracks or caries and having a separate mesial root canal such that a #10 file could pass the apical foramen while #15 K-file could not pass the apex [15].

Periapical radiographs were obtained of teeth buccolingually and mesiodistally and those with calcified canals and internal root resorption were excluded. 

The teeth were cleaned from tissue residues and debris using a scaler and a sterile gauze and were then immersed in 5.25% sodium hypochlorite solution for 1 h for disinfection. The teeth were then stored in saline until the experiment. The teeth were mounted in putty impression material (Speedex; Coltene, Altstätten, Switzerland) such that their roots were completely embedded in the impression material while their crowns were out of it. An impression of the root was obtained to stabilize the tooth position and ensure reproducibility and comparability of images [23]. To mark the mesiobuccal canal of teeth, gutta-percha was placed beside the mesiobuccal root to 1, 3 and 7 mm from the apex in order to differentiate the mesio-buccal and mesio-lingual roots. To prevent any change in anatomy of the apex, no file was introduced into the canals prior to scanning. Also, metal restorations (if any) were removed to prevent metal artifact. Next, the teeth underwent CBCT imaging (Rotograph Evo 3D; Villa Sistemi Medicali, Buccinasco MI, Italy) at 60 kVp, 6 mA and 11.2 sec time with 0.5 mm slice thickness (0.166 mm voxel size) in mesio-distal direction. The CBCT unit had 0.01 mm accuracy (Figure 1). Only teeth with 20° to 40° root curvature angle, and radius of curvature less than 4 cm were included. After initial tooth selection, canal curvature was determined on CBCT scans [15] using on Demand software (Cybermed Inc. Seoul, South Korea), transverse sections perpendicular to the longitudinal axis of the teeth were reconstructed at 1, 3 and 7 mm from the apex. Preoperative CBCT scans were obtained of samples as such by an oral and maxillofacial radiologist. 

The teeth were then taken out of the putty impression and were randomly divided into two groups (*n*=15). Stratified random sampling was used to allocate the teeth samples to each group. A #10 K-file (Dentsply Maillefer, Ballaigues, Switzerland) was then introduced into the canal until its tip was visible at the apex. Working length was determined 0.5 mm short of the apex. 

In group A, root canals were shaped by ProTaper (Dentsply Maillefer, Ballaigues, Switzerland) files using the crown down technique with a low-speed handpiece and an electric motor (NSK, Tokyo, Japan). S1, S2, F1 and F2 files at 250 rpm speed and 2.8 Ncm torque were passively used with a gentle in and out pecking motion until reaching working length.

In group B, SafeSider system (Essential Dental Systems, NJ, USA) was used for root canal shaping. The first file 25/0.06 was used passively in Endo-Express handpiece (Essential Dental Systems, NJ, USA) at 2500 rpm as recommended by the manufacturer. 

Two samples were used as controls to assess the accuracy of systems, impression technique and standardization of mounting of samples. After preoperative CBCT scanning, no preparation was performed for these two samples and postoperative CBCT scans were taken of them. To stabilize the tooth position and ensure reproducibility and comparability of images, the samples were mounted in a jig made of putty impression material as practiced in a previous study [14, 23].

In use of both systems, patency was maintained using a #10 K-file after using each rotary file. A new file was used for every five root canal preparations. After using each file, root canals were rinsed with 2.5% sodium hypochlorite solution with a 27-gauge needle and the samples were then stored in saline. For uniform preparation of apical area in terms of apical size, master apical file of #25 was used in all teeth. NaOCl was used as lubricant during preparation. All root canal preparations were performed by the same experienced endodontist.

All teeth were placed again in their respective putty impressions (used for preoperative CBCT) and CBCT scans were taken again with the same exposure settings mentioned earlier. 

Two samples were used as controls to assess the accuracy of systems, impression technique and standardization of mounting of samples. After preoperative CBCT scanning, no preparation was performed for these two samples and postoperative CBCT scans were obtained. 

Preoperative and postoperative cross-sectional views of the roots at 1, 3 and 7 mm from the apex were analyzed by the software. For each tooth, a folder was created in a computer containing one preoperative and one postoperative image showing root cross-section at 1, 3 and 7 mm from the apex (two images per each level). The distance from the internal canal wall to the external root surface was measured at the mesial and distal on preoperative and postoperative images of each level. Measurements were made by two examiners and the mean of the two values was calculated and placed in canal centering ability formula as follows: $\frac{\left(\mathrm{x1}-\mathrm{x2}\right)}{\left(\mathrm{y1}-\mathrm{y2}\right)}$ or $\frac{\left(\mathrm{y1}-\mathrm{y2}\right)}{\left(\mathrm{x1}-\mathrm{x2}\right)}$

Canal transportation was also calculated using the formula ((x_2_-x_1_)-(y_2_-y_1_)) [24] where x_1_ shows the shortest distance from the mesial root surface to mesial canal wall before preparation and x_2_ shows the shortest distance from the mesial root surface to mesial canal wall after preparation; y_1_ indicates the shortest distance from distal root surface to distal canal wall before preparation and y_2_ shows the shortest distance from distal root surface to distal canal wall after preparation (Figure 1). Next, the minimum and maximum canal centering ability were analyzed using *t*-test; if the obtained value was 1, it indicated that the file could maintain central canal path. The farther the value from 1, the greater the deviation from the original canal path. Also, the closer the canal transportation value to zero, the lower the canal transportation [24]. The *t*-test was used to compare the canal centering ability and apical transportation of the two systems while repeated measure ANOVA was used to compare the samples in each group. Also, a chronometer was used to record the mean duration of root canal preparation by each system and *t*-test was applied to compare the two groups in this respect. Only the instrumentation time was recorded.

**Table 1:** The centering ratios of the two systems at different levels from the apex

**Distance from apex**	**1 mm**	**3 mm**	**7 mm**	***P*** **-value **
**System/Centering ratio**	**Mean (SD)**	**Mean (SD)**	**Mean (SD)**	
**SafeSider**	0.24 (0.14)	0.41 (0.16)	0.58 (0.17)	*P*<0.001
**ProTaper**	0.73 (0.14)	0.83 (0.05)	0.91 (0.47)	*P*<0.001
***P*** **-value **	*P*=0.000	*P*=0.000	*P*<0.001	-

**Table 2:** Amount of canal transportation in use of the two systems at different levels from the apex

**Distance from apex**	**1 mm**	**3 mm**	**7 mm**	***P*** **-value **
**System/Canal transportation**	**Mean (SD)**	**Mean (SD)**	**Mean (SD)**	
**SafeSider**	0.56 (0.54)	0.40 (0.37)	0.23 (0.03)	*P*<0.001
**ProTaper**	0.06 (0.03)	0.03 (0.01)	0.03 (0.01)	*P*<0.001
***P*** **-value **	*P*=0.000	*P*=0.000	*P*<0.001	-

## Results

A total of 30 samples were evaluated in two groups of SafeSider and ProTaper (*n*=15). Table 1 shows changes in central canal path in designated levels from the apex by use of the two systems. 

At 1, 3 and 7 mm distance from the apex, the centering ratio of ProTaper system was higher than that of SafeSider system and this difference was statistically significant (*P*<0.001).

According to repeated measure ANOVA, in ProTaper group, canal transportation in the apical part was significantly more than coronal and middle part of the root and this association was statistically significant (*P*<0.001). 

In SafeSider group, canal transportation increased from the apical towards the coronal part of the root and this difference at different levels was statistically significant (*P*<0.001). Table 2 shows canal transportation at different levels from the apex. 

As seen in Table 2, at 1, 3 and 7 mm distance from the apex, canal transportation in SafeSider system was over 10 times greater than that in ProTaper system and this difference was statistically significant (*P*<0.001, *P*<0.001 and *P*<0.02, respectively).

The mean duration of root canal preparation was 77.77±33.44 sec by the SafeSider system and 51.08±9.01 sec by the ProTaper system. The *t*-test showed that canal preparation by ProTaper was significantly faster than that by SafeSider (*P*<0.02). 

## Discussion

This study aimed to compare ProTaper and SafeSider systems in terms of canal transportation and centering ability at 1, 3, and 7 mm distances from the apex in curved mesiobuccal root canals of mandibular first molars. 

Several NiTi files have been introduced to overcome the limitations of stainless steel hand files especially for use in curved canals. NiTi rotary files decrease the working time and procedural errors. However, they may break in curved canals [3]. Reciprocating systems were introduced to overcome the limitations of NiTi systems; however, information on the shaping ability of these systems is limited [3].

Several methods are used for assessment of the efficacy of NiTi files in maintaining original central canal path such as radiography [20], sectioning according to Bramante’s method [25], longitudinal clearing of teeth [24], high-resolution computed tomography [15, 16], micro-computed tomography [26, 27] and CBCT [14, 28]. CBCT is a high-resolution imaging modality suitable for evaluation of root canal morphology, fractures and changes in root canal system following preparation [14, 29]. CBCT was used in the current study since this non-invasive imaging modality enables accurate and reproducible three-dimensional assessment of the root canal system without damaging the samples [11, 30]. Two methods are available for measurement of canal transportation on CBCT scans. Some studies superimposed pre- and postoperative images to determine changes in canal path due to preparation [31]. Others measured the distance between the external root surface and internal canal wall at the mesial and distal aspects on pre- and postoperative cross-sectional images at three different levels. Using relevant formulas, changes in canal path were determined [32, 33]. This method was used in our study and the distance between the external root surface and internal root canal wall was measured at three levels (1, 3 and 7 mm from the apex) to determine changes in apical and middle thirds of the root canal because the risk of procedural errors is higher in these two areas. 

Mesiobuccal root of mandibular first molars with 20°to 40° curvatures were evaluated in our study because risk of ledge formation, canal transportation and perforation is higher in curved canals particularly in mesiobuccal canal of mandibular molars [34]. 

In the current study, SafeSider system caused greater canal transportation in the apical region and had a poorer canal centering ability compared to ProTaper system. Both systems caused canal transportation but SafeSider caused significantly greater canal transportation particularly at 1 mm distance from the apex and had a poorer canal centering ability (*P*<0.001). Also, in both systems from apical to coronal part, the centering ability increased while canal transport decreased only at apical part (*P*<0.001). Beveled lateral surfaces of SafeSider files have been designed to improve flexibility of these stainless-steel files [14]. Also, the back and forth motion of these files is expected to create a balanced force as the file is introduced into the root canal [19]. However, several studies have reported significant canal transportation following the use of stainless steel files with back and forth motion [35, 36]. Rhodes *et al.* [20] reported that SafeSider caused greater deviation of root canal wall compared to Vortex 06. Results of several studies have supported the superiority of NiTi instruments compared to stainless steel files in maintaining root canal curvature [35, 37]. Ceyhanli *et al.* [1] reported that canal transportation following preparation with SafeSider was higher than that with ProTaper Universal and Race, and SafeSider had poorer canal centering ability compared to the other two systems. Their study only evaluated the apical part at a 4 mm length, while this study also evaluates the middle and coronal parts. While Wigler *et al.* [38] did not find a difference in apical transportation using these two systems in apical thirds of curvature canals. The current results showed that ProTaper was well capable of maintaining the central canal path in curved canals, which was in line with the findings of other studies [39, 40]. These results may be due to the cross-sectional design of ProTaper files. These files have smaller contact area with dentin and have U-shaped grooves, which is claimed to increase the flexibility of files [41]. 

ProTaper Universal has a superior performance compared to conventional ProTaper system because the file tip has changed from a “guiding tip” to a “safe round tip” [42]. Gergi *et al.* [43] reported that canal transportation by use of this system is higher than that by use of twisted files and stainless steel hand K-files; they reported the reason to be sharp cutting edges and continuous tapering along the cutting edges of the files. 

Root canal preparation time with ProTaper system in the current study was significantly shorter than that with SafeSider. Use of a higher number of files in SafeSider system explains longer working time with this system. 

In the current study, canal transportation at 1 mm from the apex was significantly greater than that at 3 and 7 mm distances from the apex in both groups. Also, at 1 mm from the apex, files had significantly lower canal centering ability compared to the same files at 3 and 7 mm distances; this finding was in agreement with the results of Ceyhanli *et al.* [1] that showed SafeSider and ProTaper caused greater canal transportation at 1 mm from the apex compared to other levels. Wu *et al.* [44] reported that apical transportation greater than 0.3 mm decreases the quality of apical seal. Our results showed that SafeSider system exceeded this critical threshold, which calls for further studies on this system. However, in accordance with the study by Berutti *et al.* [45], ProTaper did not exceed the critical threshold in our study.

Future studies are required to assess the efficacy of SafeSider system in other teeth with variable degrees of root curvature in comparison with other rotary systems with reciprocating motion. Also, SafeSider system must be further evaluated in terms of crack formation, dentin removal and file fracture.

## Conclusion

Within the limitations of this study, the results showed that ProTaper system (in contrast to SafeSider) was well capable of preparation of narrow curved canals with high canal centering ability causing minimal transportation. In both systems, canal centering ability decreased and canal transportation increased from the coronal towards the apical region. Working time with ProTaper was significantly shorter than that with SafeSider. 

## References

[1] Ceyhanli KT, Erdilek N, Tatar I, Cetintav B (2014). Comparative micro-computed tomography evaluation of apical root canal transportation with the use of ProTaper, RaCe and Safesider systems in human teeth. Aust Endod J.

[2] Schilder H (1974). Cleaning and shaping the root canal. Dent Clin North Am.

[3] Abou-Rass M, Frank AL, Glick DH (1980). The anticurvature filing method to prepare the curved root canal. J Am Dent Assoc.

[4] Guelzow A, Stamm O, Martus P, Kielbassa AM (2005). Comparative study of six rotary nickel-titanium systems and hand instrumentation for root canal preparation. Int Endod J.

[5] Gluskin AH, Brown DC, Buchanan LS (2001). A reconstructed computerized tomographic comparison of Ni-Ti rotary GT files versus traditional instruments in canals shaped by novice operators. Int Endod J.

[6] Pruett JP, Clement DJ, Carnes DL Jr (1997). Cyclic fatigue testing of nickel-titanium endodontic instruments. J Endod.

[7] Haikel Y, Serfaty R, Bateman G, Senger B, Allemann C (1999). Dynamic and cyclic fatigue of engine-driven rotary nickel-titanium endodontic instruments. J Endod.

[8] Ruddle C (2001). The ProTaper technique: endodontics made easier. Dentistry today.

[9] Ruddle CJ (2001). The ProTaper endodontic system: geometries, features, and guidelines for use. Dentistry today.

[10] Tambe VH, Nagmode PS, Abraham S, Patait M, Lahoti PV, Jaju N Comparison of canal transportation and centering ability of rotary protaper, one shape system and wave one system using cone beam computed tomography: An in vitro study.

[11] Madani Z, Soleymani A, Bagheri T, Moudi E, Bijani A, Rakhshan V Transportation and Centering Ability of Neoniti and ProTaper Instruments; A CBCT Assessment.

[12] Varela-Patino P, Ibanez-Parraga A Fau - Rivas-Mundina B, Rivas-Mundina B Fau - Cantatore G, Cantatore G Fau - Otero XL, Otero Xl Fau - Martin-Biedma B, Martin-Biedma B Alternating versus continuous rotation: a comparative study of the effect on instrument life.

[13] Lopes HP, Elias Cn Fau, Vieira MVB, Vieira Mv Fau, Siqueira JF Jr, Siqueira Jf Jr Fau, Mangelli M, Mangelli M Fau, Lopes WSP, Lopes Ws Fau, Vieira VTL, Vieira Vt Fau, Alves FRF, Alves Fr Fau, Oliveira JCM, Oliveira Jc Fau, Soares TG, Soares TG Fatigue Life of Reciproc and Mtwo instruments subjected to static and dynamic tests.

[14] Setzer FC, Kwon TK, Karabucak B (2010). Comparison of apical transportation between two rotary file systems and two hybrid rotary instrumentation sequences. J Endod.

[15] Estrela C, Bueno MR, Sousa-Neto MD, Pecora JD (2008). Method for determination of root curvature radius using cone-beam computed tomography images. Braz Dent J.

[16] McRay B, Cox TC, Cohenca N, Johnson JD, Paranjpe A (2014). A micro-computed tomography-based comparison of the canal transportation and centering ability of ProTaper Universal rotary and WaveOne reciprocating files. Quintessence Int.

[17] Bernardes RA, Rocha EA, Duarte MA, Vivan RR, de Moraes IG, Bramante AS, de Azevedo JR (2010). Root canal area increase promoted by the EndoSequence and ProTaper systems: comparison by computed tomography. J Endod.

[18] You SY, Bae KS, Baek SH, Kum KY, Shon WJ, Lee W (2010). Lifespan of one nickel-titanium rotary file with reciprocating motion in curved root canals. J Endod.

[19] De‐Deus G, Moreira E, Lopes H, Elias C (2010). Extended cyclic fatigue life of F2 ProTaper instruments used in reciprocating movement. International endodontic journal.

[20] Rhodes SC, Hulsmann M, McNeal SF, Beck P, Eleazer PD (2011). Comparison of root canal preparation using reciprocating Safesiders stainless steel and Vortex nickel-titanium instruments. Oral Surg Oral Med Oral Pathol Oral Radiol Endod.

[21] You SY, Kim HC, Bae KS, Baek SH, Kum KY, Lee W (2011). Shaping ability of reciprocating motion in curved root canals: a comparative study with micro-computed tomography. J Endod.

[22] Honardar K, Assadian H, Shahab S, Jafari Z, Kazemi A, Nazarimoghaddam K, Kharrazifard MJ, Labbaf H (2014). Cone-beam Computed Tomographic Assessment of Canal Centering Ability and Transportation after Preparation with Twisted File and Bio RaCe Instrumentation. J Dent (Teh).

[23] Khademi A, Yazdizadeh M, Feizianfard M (2006). Determination of the minimum instrumentation size for penetration of irrigants to the apical third of root canal systems. J Endod.

[24] Barthel CR, Gruber S, Roulet JF (1999). A new method to assess the results of instrumentation techniques in the root canal. J Endod.

[25] Bramante CM, Berbert A, Borges RP (1987). A methodology for evaluation of root canal instrumentation. J Endod.

[26] Shokouhinejad N, Nekoofar MH, Iravani A, Kharrazifard MJ, Dummer PM (2010). Effect of acidic environment on the push-out bond strength of mineral trioxide aggregate. J Endod.

[27] Gao Y, Peters OA, Wu H, Zhou X (2009). An application framework of three-dimensional reconstruction and measurement for endodontic research. J Endod.

[28] Madani ZS, Haddadi A, Haghanifar S, Bijani A Cone-beam computed tomography for evaluation of apical transportation in root canals prepared by two rotary systems.

[29] Wenzel A, Haiter-Neto F, Frydenberg M, Kirkevang LL (2009). Variable-resolution cone-beam computerized tomography with enhancement filtration compared with intraoral photostimulable phosphor radiography in detection of transverse root fractures in an in vitro model. Oral Surg Oral Med Oral Pathol Oral Radiol Endod.

[30] Hashem AA, Ghoneim AG, Lutfy RA, Foda MY, Omar GA (2012). Geometric analysis of root canals prepared by four rotary NiTi shaping systems. J Endod.

[31] Kaya S, Yigit Ozer S, Adiguzel O, Orucoglu H, Deger Y, Tumen EC, Uysal I (2015). Comparison of apical microleakage of dual-curing resin cements with fluid-filtration and dye extraction techniques. Med Sci Monit.

[32] Gambill JM, Alder M, del Rio CE (1996). Comparison of nickel-titanium and stainless steel hand-file instrumentation using computed tomography. J Endod.

[33] Freire LG, Gavini G, Branco-Barletta F, Sanches-Cunha R, dos Santos M (2011). Microscopic computerized tomographic evaluation of root canal transportation prepared with twisted or ground nickel-titanium rotary instruments. Oral Surg Oral Med Oral Pathol Oral Radiol Endod.

[34] Tasdemir T, Aydemir H, Inan U, Unal O (2005). Canal preparation with Hero 642 rotary Ni-Ti instruments compared with stainless steel hand K-file assessed using computed tomography. Int Endod J.

[35] Paque F, Barbakow F, Peters OA (2005). Root canal preparation with Endo-Eze AET: changes in root canal shape assessed by micro-computed tomography. Int Endod J.

[36] Frank AL (1967). An evaluation of the Giromatic endodontic handpiece. Oral Surg Oral Med Oral Pathol.

[37] Hulsmann M, Schade M, Schafers F (2001). A comparative study of root canal preparation with HERO 642 and Quantec SC rotary Ni-Ti instruments. Int Endod J.

[38] Wigler R, Koren T, Tsesis I Evaluation of Root Canal Cleaning and Shaping Efficacy of Three Engine-driven Instruments: SafeSider, ProTaper Universal and Lightspeed LSX.

[39] Duran-Sindreu F, Garcia M, Olivieri JG, Mercade M, Morello S, Roig M (2012). A comparison of apical transportation between FlexMaster and Twisted Files rotary instruments. J Endod.

[40] Huang X, Ling J, Wei X, Gu L (2007). Quantitative evaluation of debris extruded apically by using ProTaper Universal Tulsa rotary system in endodontic retreatment. J Endod.

[41] Versiani MA, Leoni GB, Steier L, De-Deus G, Tassani S, Pecora JD, de Sousa-Neto MD (2013). Micro-computed tomography study of oval-shaped canals prepared with the self-adjusting file, Reciproc, WaveOne, and ProTaper universal systems. J Endod.

[42] Camara AS, de Castro Martins R, Viana AC, de Toledo Leonardo R, Buono VT, de Azevedo Bahia MG (2009). Flexibility and torsional strength of ProTaper and ProTaper Universal rotary instruments assessed by mechanical tests. J Endod.

[43] Gergi R, Rjeily JA, Sader J, Naaman A (2010). Comparison of canal transportation and centering ability of twisted files, Pathfile-ProTaper system, and stainless steel hand K-files by using computed tomography. J Endod.

[44] Wu MK, Fan B, Wesselink PR (2000). Leakage along apical root fillings in curved root canals Part I: effects of apical transportation on seal of root fillings. J Endod.

[45] Berutti E, Chiandussi G, Paolino DS, Scotti N, Cantatore G, Castellucci A, Pasqualini D (2012). Canal shaping with WaveOne Primary reciprocating files and ProTaper system: a comparative study. J Endod.

